# Divergent Effects of the Nonselective Adenosine Receptor Antagonist Caffeine in Pre-Manifest and Motor-Manifest Huntington’s Disease

**DOI:** 10.3390/biomedicines10061258

**Published:** 2022-05-27

**Authors:** Jannis Achenbach, Andreas Matusch, David Elmenhorst, Andreas Bauer, Carsten Saft

**Affiliations:** 1 Department of Neurology, Huntington Center North Rhine-Westphalia, St. Josef-Hospital Bochum, Ruhr-University Bochum, Gudrunstraße 56, 44791 Bochum, Germany; carsten.saft@rub.de; 2 Institute of Neuroscience and Medicine (INM-2), Forschungszentrum Jülich, Wilhelm-Johnen-Straße, 52428 Jülich, Germany; a.matusch@fz-juelich.de (A.M.); d.elmenhorst@fz-juelich.de (D.E.); an.bauer@fz-juelich.de (A.B.); 3 Department of Nuclear Medicine, Faculty of Medicine and University Hospital Cologne, University of Cologne, 50923 Cologne, Germany

**Keywords:** Huntington’s Disease, adenosine receptor antagonist, caffeine, coffee consumption, ENROLL-HD

## Abstract

There is a controversy about potentially positive or negative effects of caffeine consumption on onset and disease progression of neurodegenerative diseases such as Huntington’s Disease (HD). On the molecular level, the psychoactive drug caffeine targets in particular adenosine receptors (AR) as a nonselective antagonist. The aim of this study was to evaluate clinical effects of caffeine consumption in patients suffering from premanifest and motor-manifest HD. Data of the global observational study ENROLL-HD were used, in order to analyze the course of HD regarding symptoms onset, motor, functional, cognitive and psychiatric parameters, using cross-sectional and longitudinal data of up to three years. We split premanifest and manifest participants into two subgroups: consumers of >3 cups of caffeine (coffee, cola or black tea) per day (>375 mL) vs. subjects without caffeine consumption. Data were analyzed using ANCOVA-analyses for cross-sectional and repeated measures analysis of variance for longitudinal parameters in IBM SPSS Statistics V.28. Within *n* = 21,045 participants, we identified *n* = 1901 premanifest and *n* = 4072 manifest HD patients consuming >3 cups of caffeine/day vs. *n* = 841 premanifest and *n* = 2243 manifest subjects without consumption. Manifest HD patients consuming >3 cups exhibited a significantly better performance in a series of neuropsychological tests. They also showed at the median a later onset of symptoms (all *p* < 0.001), and, during follow-up, less motor, functional and cognitive impairments in the majority of tests (all *p* < 0.050). In contrast, there were no beneficial caffeine-related effects on neuropsychological performance in premanifest HD mutation carriers. They showed even worse cognitive performances in stroop color naming (SCNT) and stroop color reading (SWRT) tests (all *p* < 0.050) and revealed more anxiety, depression and irritability subscores in comparison to premanifest participants without caffeine consumption. Similarly, higher self-reported anxiety and irritability were observed in genotype negative/control group high dose caffeine drinkers, associated with a slightly better performance in some cognitive tasks (all *p* < 0.050). The analysis of the impact of caffeine consumption in the largest real-world cohort of HD mutation carriers revealed beneficial effects on neuropsychological performance as well as manifestation and course of disease in manifest HD patients while premanifest HD mutation carrier showed no neuropsychological improvements, but worse cognitive performances in some tasks and exhibited more severe signs of psychiatric impairment. Our data point to state-related psychomotor-stimulant effects of caffeine in HD that might be related to regulatory effects at cerebral adenosine receptors. Further studies are required to validate findings, exclude potential other unknown biasing factors such as physical activity, pharmacological interventions, gender differences or chronic habitual influences and test for dosage related effects.

## 1. Introduction

Neurodegenerative autosomal-dominant inherited Huntington’s Disease (HD) caused by a CAG-trinucleotide repeat expansion in the huntingtin gene (*HTT*) is characterized by manifold serious motor, cognitive and psychiatric symptoms [1,2]. Complex mechanisms involve mutant *HTT* (*mHTT*) disrupting transcription, immunological and mitochondrial dysfunction and pathogenic somatic CAG expansion in vulnerable cells as part of pathogenic molecular mechanisms [3,4,5].

Although there are manifold recent and promising therapeutic approaches such as gene therapies, mRNA modulating targets using antisense oligonucleotides or small molecules, until now, no disease modifying or causal therapy is available [6,7,8,9]. Lacking causative treatment options, commonly used symptomatic pharmacological treatments or lifestyle-based effects were analyzed with regard to potential influences on disease manifestation and progression of HD [10,11]. The ENROLL-HD worldwide observational study is designed as a clinical research platform, providing real-world data of HD patients, premanifest mutation carriers and controls and an ideal starting point for investigation of these issues [12]. Here, we aimed to analyze caffeine-consumption in premanifest and manifest affected HD patients to evaluate disease manifestation, progression and potential beneficial or worsening effects.

Epidemiological evidence verifies human caffeine consumption as the most widely consumed psychoactive drug. The mechanism of action is the nonselective adenosine receptor (AR) antagonization, postulated to positively reduce cognitive decline in Alzheimer’s (AD) and Parkinson’s Disease (PD) [13,14]. 

Caffeine has “multi-benefits” in PD, describing an observed relationship between decreasing risks for developing PD associated with increased caffeine consumption in epidemiological data as well as neuroprotective, motor and cognitive benefits in PD models [15]. Caffeine-consumption as a modifiable environmental factor is furthermore discussed to have a positive effect on the risk of developing AD or age-related cognitive impairment [16]. Although chronic caffeine exposure through consumption of tea or coffee was investigated in former reviews and identified to have beneficial effects on cognitive capacities, mood in preclinical and clinical interventions and neuroprotective properties on dopaminergic neurons, the link between underlying roles of caffeine on neurotransmitters and neurobehavioral effects is not fully understood [17,18,19]. Next to caffeine, further bioactive components such as chlorogenic acid, caffeic acid, trigonelline, kahweol, and cafestol are discussed to play an unknown neuroprotective role in coffee beverages [20].

Using preclinical models, the nematode *Caenorhabditis elegans* (*C. elegans*) was assessed as an animal model in manifold neurodegenerative disorders not only to evaluate effects of pharmacological or nutraceutical interventions and to identify neuroprotective candidates in neuronal cells, but also to gain insights into so-called “behavioral genetics” [21,22]. Manifold former research investigated different approaches of HD in *C. elegans* to investigate therapeutic effects of potential disease modifying targets or to identify underlying pathophysiological neurotoxic cascades [23,24,25]. Hereby, caffeine was identified as a relevant factor to extend life span and improve health span in *C. elegans* delaying pathology in the nematode model of the polyglutamine disease suggesting chronic consumption enhances resistance to proteotoxic stress and is a relevant target to treat human Alzheimer’s (AD) or Huntington’s Disease [26]. Additionally, other preclinical models investigated caffeine having neuroprotective effects in cerebrospinal ataxia 3 as another abnormal CAG-repeat expansion and polyglutamine- related disease, which might be relevant for HD [27]. 

Further research investigated adenosine receptors (AR) and the role of their subtypes A_1_, A_2A_, A_2B_, and A_3_ in pathophysiological processes of HD [28]. Caffeine in the overall context of interactions with Adenosine and brain functions [29], and as a relevant substance mainly affecting A_1_ and A_2A_ AR subtypes as a target [30], was postulated to be positively associated with the age of HD onset [28].

In addition, genetic polymorphism of the A_2A_ AR may impact the age of onset- identifying adenosine receptors as potential targets for pharmaceutical treatment approaches or as helpful biomarkers in HD [28,31,32,33]. In humans, so far, investigation of A_1_ AR is possible by using the radioligands [^18^F]CPFPX or [^11^C]MPDX and positron emission tomography (PET) in subjects pausing caffeine consumption [34]. A_2A_ AR was imaged in humans using [^11^C]TMSX, [^11^C]SCH442416, [^18^F]FESCH, and [^18^F]MNI-444 reviewed by Lai and coworkers [35]. The first study investigating these receptors in HD described higher cerebral A_1_ AR values in premanifest HD subjects, followed by a continuous decrease and reduced levels especially in the caudate and amygdala in manifest HD if compared to controls supporting the important role of adenosine receptors in the pathophysiology of HD [36]. Besides HD, adenosine and ARs were investigated as modulators to positively influence cognitive functions due to neuromodulation (synaptic plasticity) and homeostatic functions in brain regions modulating dopamine, glutamate, and brain-derived neurotrophic factor (BDNF) signaling metabolism [13]. Experimental animal models of HD suggested caffeine as A_2_A receptor antagonist having antioxidant, neuroprotective effects reducing mitochondrial dysfunction and oxidative stress [37].

In contrast, more recently, Wang et al. reported genetic variants associated with higher coffee consumptions and an earlier HD onset [38]. This is a confirmation of earlier clinical research assessed caffeine as a life-style modifier in HD within *n* = 80 patients suggesting consumptions greater than 190 mg/day significantly associated with earlier age of onset [39]. Thus, a dosage of caffeine consumption might be relevant [39]. Because various disease patterns and variances in age of onset (AO) are known in HD postulating that approximately 60% of the AO variance is not explainable due to the CAG-load in the HD mutation, there is an urgent need to identify modulating pharmacological or lifestyle-based effects such as caffeine-consumption [40]. The conundrum of positive effects in other neurodegenerative diseases, positive effects in HD animal models on molecular mechanisms and recent investigations describing hints with earlier HD onsets in humans have huge implications for affected patients because coffee consumption is widely common and easily influenceable. 

Until now, coffee drinking habits in affected HD patients and potential effects in large real-world cohorts have not been investigated. This research aimed to evaluate caffeine-consumption within the worldwide largest research HD cohort and to depict effects on disease onset, motor, cognitive, functional and psychiatric disease manifestation as well as progression. For comparison of data with those not affected by HD, we in addition analyzed data from family controls and gene negative subjects coming from this study.

## 2. Methods

### 2.1. Investigating the ENROLL-HD Database

We analyzed the ENROLL-HD periodic dataset five to investigate the habitual caffeine-consumption in premanifest, manifest HD patients and family controls to depict potential effects on the course of disease if compared to participants without any caffeine-consumption. Enroll-HD is a global clinical research platform designed to facilitate clinical research in HD. Core datasets are collected annually from all research participants as part of this global multi-center longitudinal observational study. Data are monitored for quality and accuracy using a risk-based monitoring approach. All sites are required to obtain and maintain local ethical approval. We investigated the periodic dataset five (PDS5) as previously described [10,41]. Ethics approval was obtained by the local ethics committee of Ruhr-University Bochum (No. 4941-14).

Participants were categorized into premanifest and motor-manifest groups as well as family controls/genotype negative HD and subdivided into participants without any caffeine consumption and >3 cups/day (at least >375 mL) (coffee, black tea or cola) as assessed within the baseline data of study entry in a cross-sectional approach. We excluded participants with <3 cups/day in order to achieve a clear stratification of the participants.

As inclusion criteria for the manifest HD group, we analyzed all participants with a diagnostic confidence level (DCL) of 4 (having unequivocal signs of clinical manifest HD (>99% confidence), a total motor-score (TMS) >5 and a genetically confirmed report with ≥36 Cytosine–Adenine–Guanine (CAG)-repeats in the Huntingtin-gene (*HTT*). Longitudinal data with annual (±3 months) follow-up visits of up to three more years were analyzed to compare disease manifestation and progression over time.

Fundamental demographic and genetic parameters were assessed analyzing CAG-repeat lengths, age, sex, educational level, age at HD diagnosis, age at onset of symptoms reported by the patient, family and rater between groups. We calculated the CAG-Age Product-Index (CAP-Score) as an index for the disease burden [42]. As an additional explorative approach (results added as Supplementary Materials), we calculated prognostic years to symptom onset for pre-manifest cohorts as reported earlier [43]. We therewith compared cognitive symptoms between non-caffeine consumers and those drinking more than 3 cups of caffeine/day grouped by prognosed onsets (<5, 5– < 10, 10– < 15, 15– < 20 and >20 years to onset). Grouped analysis according to disease stages were conducted as additional explorative analysis for manifest patients as stage 1 (TFC 13-11), stage 2 (TFC 10-7), stage 3 (TFC 6-3), stage 4 (TFC 2-1) and stage 5 (TFC 0) to estimate influencing factors according to progression in neurodegeneration [44,45].

Motor parameters were analyzed using the UHDRS-Total motor score. Cognitive performance was evaluated with the ENROLL-HD test battery including six cognitive tests: Symbol digit modality test (SDMT), Verbal fluency test (category; VFc), Stroop color naming (SCNT), Stroop-word reading (SWRT), Stroop interference test (SIT) and Mini mental state examination (MMSE). Functionality was analyzed within the UHDRS-Total functional capacity (TFC) and Independence Scale (IS). Psychiatric behavior was analyzed between groups using assessments reported by the clinical rater within the Problem Behaviours Assessment–Short (PBA-s) questionnaire with subscores for depression, irritability/aggression, psychosis, apathy, executive function and self-reported assessments using the Hospital Anxiety and Depression Scale (HADS-IS)-questionnaire with subscores for depression, irritability, outward and inward irritability.

### 2.2. Statistical Analyses

We analyzed group means and standard deviation at baseline visit as cross-sectional data using univariate analysis of variance (ANCOVA) controlling for co-variates age, CAG-repeat length and education. Dependent variables were tested for normal distribution using the Kolmogorov–Smirnov test (data not shown). Homogeneity of variances was asserted using Levene’s Test. Unequal variances were further assessed with Welch’s *t*-test. Chi-square tests were used for analyses of categorical variables. We compared disease manifestation between groups in IBM SPSS Statistics V.28. Longitudinal analyses were calculated for motor, functional and cognitive parameters using repeated measures analysis of variance between groups at baseline and three more follow up visits.

## 3. Results

### 3.1. Participants and Data Analyses

**Figure 1:**
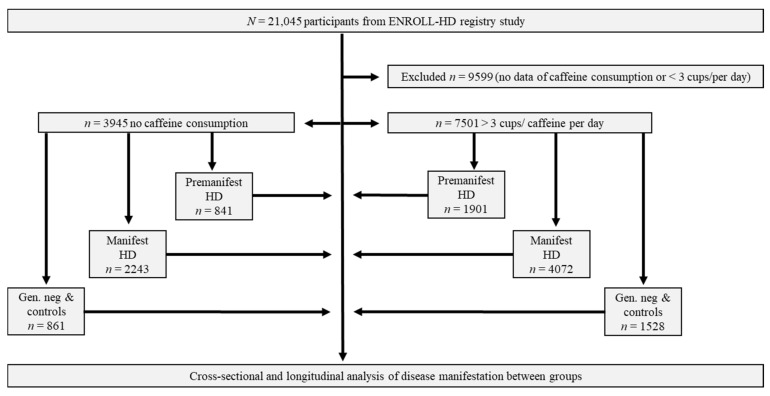
Workflow of analysis: Premanifest, manifest and control-participants from ENROLL-HD divided due to caffeine consumption. Abbreviations: n/N, Number; PDS-5, Periodic dataset 5; HD, Huntington’s Disease.

Within the Enroll-HD periodic dataset five, out of *n* = 21,116 participants, *n* = 7051 revealed to drink more than three cups of caffeine per day as stated within their clinical assessments. *n* = 1901 out of these 7501 participants appeared to be premanifest and *n* = 4072 manifest HD patients. As control groups, we identified *n* = 841 premanifest and *n* = 2243 manifest HD patients without any coffee consumption. Cohorts of *n* = 1528 family controls with consumption of >3 cups of caffeine per day and *n* = 861 family members without coffee consumption were excluded within further analysis (Figure 1).

### 3.2. Comparison of Premanifest HD Participants

Assessing cross-sectional data between premanifest HD groups revealed differences in fundamental demographic parameters. We therefore controlled for further analysis with co-variates age, CAG-repeat length and education. Motor, functional and four (SDMT, VFc, SIT, MMSE) out of six cognitive tests of the cognitive test battery in the ENROLL-HD database revealed no statistically significant differences between premanifest HD groups drinking >3 cups of caffeine/day and those without any consumption. The Stroop color naming and Stroop word reading test as part of the cognitive test-battery showed that premanifest patients without consumption performed significantly better during baseline assessment (all *p* < 0.05). With regard to psychiatric parameters, premanifest HD with >3 cups/day revealed significantly more depression and irritability within the PBA, as investigator-assessed, and self-reported higher anxiety, depression and irritability within HADS-IS scales (all *p* < 0.05; Table 1). As an additional explorative approach premanifest cohorts were analyzed according to their prognosed symptom onsets (Supplementary Table S1).

### 3.3. Longitudinal Data of Premanifest HD Mutation Carriers

Having established cross-sectional baseline data of premanifest participants, we analyzed longitudinal data regarding three more annual follow-up visits of study participation. Comparison of mean CAG-repeat lengths revealed 42.6 (2.7) in premanifest participants without caffeine consumption and 42.1 (2.7) in those consuming more than three cups/day. Premanifest patients without caffeine consumption were in mean 38.9 (12.0) years old, those with caffeine consumption revealed a mean age of 43.3 (11.5) years. With regard to motor, functional and cognitive testing, no group differences were observed between premanifest HD without and >3 cups of caffeine/day- consumption revealing the same performances over time. An exception was identified within the Stroop word reading test, revealing less cognitive decrease over time within premanifest HD without caffeine consumption (*p* < 0.05; Table 2).

### 3.4. Comparison of Manifest HD Participants

Next, we compared manifest HD participants drinking more than three cups of caffeine/day (*n* = 4071) vs. affected patients without any consumption (*n* = 2242), again controlling for the fundamental parameters of age, CAG-repeat length and education as co-variates. Of note, the manifest HD group with caffeine consumption revealed in mean slightly later HD diagnosis and onsets of motor symptoms as well as a later onset estimation of the clinical rater, participant and family (all *p* < 0.001). Mean data for motor (TMS), functional (TFC, IS) and cognitive performances within a test battery of six cognitive tests revealed significantly worse values within the manifest HD group without caffeine consumption (all *p* < 0.001). We then set out to analyze psychiatric disease manifestation and found that manifest HD without caffeine consumption revealed significantly higher scores for apathy and loss of executive functions within the PBA and reported about more self-assessed depression within the HADS-IS (all *p* < 0.005). Analogous with findings within the premanifest cohorts, HD participants drinking more than three cups of caffeine/day reported about higher anxiety and irritability sub-scores within the HADS-IS (all *p* < 0.050; Table 3). To assess influencing factors of neurodegeneration and disease progression in more detail, cognitive capacities were exploratively compared in manifest patients without caffeine consumption vs. those with >3 cups/day according to different disease stages (Supplementary Materials Table S2).

### 3.5. Longitudinal Data of Manifest HD Participants

Longitudinal repeated measures analysis of variance revealed manifest HD groups drinking more than three cups of caffeine/day and those without coffee consumption had different course of diseases over time with regard to motor, functional and cognitive manifestations (all *p* < 0.001). Here, we analyzed the longitudinal progression over three years within cohorts of *n* = 522 vs. *n* = 1120 participants, implemented in follow-up visits of ENROLL-HD study participation. The group of manifest patients having three more follow-up visits without caffeine consumption revealed a mean CAG-repeat length of 44.5 (4.3), an AO at the age of 44.8 (12.6) and was in mean 52.4 (12.9) years old. Manifest patients drinking more than three cups/day had a mean CAG-repeat length of 43.8 (3.4), an AO at the age of 45.4 (11.0) and an age of 51.8 (11.3) years. Longitudinally, manifest HD with more than three cups caffeine consumption/day declined less in motor (TMS), functional (TFC, IS) and cognitive capacities in five out of six tests over time (all *p* < 0.001). An exception was identified within the Verbal fluency test (Category) whereby participants without coffee consumption remained in medium more stable over time if compared to participants with coffee consumption (*p* < 0.001; Table 4). Both groups revealed decreasing motor, cognitive and functional parameters over time as depicted in descriptive mean values between baseline and follow-up three visit.

### 3.6. Family Controls and Genotype Negative Participants within the Database

As a further approach, we assessed clinical data of genotype negative and family controls consuming more than three cups of caffeine/day vs. those without any consumption to depict differences in participants not affected by HD. Similar to baseline data of premanifest and motor-manifest HD, participants with higher caffeine consumption revealed significantly more irritability and anxiety within the HADS-IS sub domains (all *p* < 0.05). As seen in manifest HD patients, participants consuming >3 cups of caffeine/day revealed significantly less impairment within the UHDRS-TFC and -IS as functional measurements (all *p* < 0.001) and better cognitive performances within the SDMT, VerFc and SCNT (all *p* < 0.050; Table 5).

Regarding longitudinal data, repeated measures analysis of variance between genotype negative and family controls consuming >3 u caffeine/day vs. those without any caffeine consumption revealed no statistically differences between groups for motor, cognitive and functional manifestation over time (data not shown). 

## 4. Discussion

Caffeine-containing beverages as coffee, tea and cola are frequently consumed due to their positive effects on vigilance and attention. Caffeine is a nonselective AR antagonist and psychoactive drug that improves impaired vigilance, most efficiently during increased sleepiness [13,14,46]. Former research investigated complex underlying mechanisms on cognitive performance within healthy subjects and identified caffeine as “the most popular neurostimulant worldwide” [47]. As described for neurodegenerative diseases such as PD and AD, beneficial effects were observed in cognitive performances. There was some controversy on data in HD, showing a negative influence on the age of onset in those having genetically predicted higher coffee consumption [38] and worsening effects caused by higher dosages of caffeine [39]. Hence, there remains an urgent need to investigate if caffeine consumption exerts positive or negative effects in HD. We therefore set out to investigate the largest cohort of pre- and motor-manifest HD mutation carriers worldwide within the ENROLL-HD registry study to compare clinical data with regard to onset, cognition, functionality and motor aspects of both patients with and without high chronic caffeine consumption.

Of note, in motor manifest HD subjects, considerable differences were observed between subjects drinking none and high caffeine drinkers. Our data revealed better motor, cognitive and functional performances in motor-manifest HD patients with extensive caffeine consumption if compared to those without any caffeine use. A potentially positive effect of higher caffeine consumption on cognitive performance was likewise observed in controls going along with former research proclaiming caffeine having positive effects on vigilance and attention, but increased irritability and anxiety. 

The molecular mechanisms explaining changes of cognitive performances and the impact on memory due to caffeine consumption are complex and depend on patterns of fatigue, sleep, habituation and earlier caffeine consumption [19,46]. To validate findings of better performances in manifest HD with high caffeine consumption, we exploratively compared cognitive capacities according to different disease stages to depict potential effects of progressed neurodegeneration. This analysis confirmed a beneficial effect of caffeine consumption in patients suffering from manifest HD, independent of the disease stage with even more pronounced effects in advanced stages. A possible explanation of better cognitive and motor performances of manifest HD patients with high and frequent caffeine consumption might be the regulation of pathophysiological functions via adenosine receptors as investigated earlier [28,48,49]. 

Additionally, interactions with symptomatic- pharmacological treatments commonly prescribed in manifest HD might contribute to the observed effect. Earlier investigations documented frequent use of neuroleptics and antipsychotic drugs as part of the medical history, partly accompanied with potential side effects [10,50]. Caffeine may partially counteract neuroleptics on striatal D_2_/A_2A_ receptor heterodimers, as D_2_ are coupled to inhibitory and A_2A_ to stimulatory G-proteins and both neuroleptics and caffeine are antagonists on D_2_ and A_2A_, respectively [51]. Our finding of more beneficial effects of caffeine consumption in the advanced HD stages—medicated most likely with higher dosages of neuroleptic pharmacotherapies—supports this potential explanation. Investigations should therefore assess a neuroleptic dose-equivalent as covariate in HD cohorts. This drug interaction might also contribute to the observed beneficial longitudinal effects with less functional and cognitive decline. Remarkably, manifest HD caffeine consumers had a slightly later HD diagnosis and onsets of symptoms versus non-consumers. Suggestions and evidence of an earlier HD onset due to caffeine-consumption as seen in former research is, therewith, not supported by the investigated ENROLL-HD real-world data [38,39]. 

### Limitations

As a limitation, our data does not allow a subdivision of individuals within the analyzed cohorts of those carrying genetically caffeine sensitive genotypes, such as homozygous C-allele carriers of ADORA_2_A (encoding adenosine A_2_A receptors) potentially being more vulnerable for observed effects [46]. For these distinctions and to validate our findings, further research with molecular genetic testing or functional imaging is necessary. As a further limitation of the present study, detailed information about exact dosages of caffeine-consumption was not available, since respective data were not assessed in the ENROLL-HD dataset. Further questionnaires could help to assess detailed dosages and consuming behaviors and should therefore be implemented in the database. Furthermore, these interventions should aim to exclude potential other unknown biasing factors such as physical activity, pharmacological interventions, gender differences or chronic habitual influences and test for dosage related effects.

In contrast to manifest HD, comparing premanifest HD mutation carriers drinking >3 cups of caffeine/day versus those without consumption revealed no differences in motor and functional performances as well as similar decrease of cognitive parameters over time. Most cross-sectional cognitive parameters revealed similar capacities and differences with impaired performance in the high caffeine consumption group in only two tests (SCNT, SWRT). According to the explorative analysis conducted with regard to the estimated age of disease onset, one might hypothesize that these slight differences were based on better cognition in those with an estimated onset beyond 10 years without caffeine consumption or a slightly negative effect of caffeine consumption, as no statistically differences were observed in patients with a predicted onset <10 years. Presumed differences in expression of adenosine A_1_ receptors in PET studies during the course of disease with higher levels and hyperstimulation even before the motor onset might be a potential explanation of slightly observed negative effects [36]. Premanifest caffeine drinkers additionally revealed more pronounced depression and irritability scores. Similarly, higher self-reported anxiety and irritability were observed in high dose caffeine drinkers within manifest HD and genotype negative/control groups. The finding of an higher self-reported depressive mood is contrary to earlier research, showing that caffeine consumption is associated with a decreased risk for depression [52]. This has also been observed in controlled animal models of repeated stress-induced depressive-like behavior [53] providing causal rather than correlative evidence of an impact of caffeine on depressive-like behavior. Higher self-reported anxiety and irritability were observed in the high dose caffeine drinkers genotype negative/control group and in the premanifest HD group. This is in line with earlier research describing higher habitual coffee consumption has an impact on brain functional connectivity going along with emotional implications [54]. 

Whilst high dosage caffeine consumption in the genotype negative/control group was associated with a slightly better performance in some cognitive tasks, it was associated with an impaired performance in some tasks in the premanifest HD group, supporting the assumption of different underlying molecular mechanisms in premanifest HD mutation carriers as discussed above. A potential explanation for the occurrence of more severe psychiatric impairments in terms of depression within the cohort of premanifest patients is the comparatively high dosage (>3 cups/day) found in this study, exceeding doses for which positive effects were described in literature [55,56]. 

Divergent effects of caffeine on cognition in premanifest versus motor-manifest HD might also be related to temporal and state-dependent adenosine receptor A_1_ AR availability in HD. Previous reports [29] revealed an increase of cerebral A_1_ AR binding potential in premanifest HD subjects versus healthy controls, followed by a continuous decrease in the following course of disease resulting in below-pre-HD and below-control A_1_AR levels in manifest HD. Beyond that, together with the A_1_ AR variation, it has been reported that the A_2_ AR density is also affected in the basal ganglia [48] and hippocampus [57] of HD animal models. Hereafter, there is even greater evidence associating caffeine and A_2_ A receptors, rather than A_1_ receptors to modify neurodegenerative processes and neuronal dysfunction [58]. Other research regarding interactions of caffeine consumption and sleep quality suggests that shorter sleep duration is associated with higher caffeine consumption as well as subjects with reduced sleep quality had greater caffeine consumption [59]. Excessive consumption is known to cause so-called “caffeine-induced sleep disorder”, additionally explaining negative effects on sleep quality in the night accompanied with daytime fatigue. This might in turn be compensated by higher caffeine intake and result in net cognitive and psychiatric impairments, as shown by others and us in premanifest cohorts [56,60]. However, as a limitation of observational in contrast to prospective studies, it is hard to distinguish whether caffeine consumption behavior is a trait marker introducing selection bias, namely between high- and low responders. This bias would then also extend into longitudinal change [61]. These limitations have been addressed in earlier research in the context of functional or sleep-quality outcomes, even in prospective human studies [62]. We cannot fully exclude other biasing factors of e.g., habitual consuming-differences in pre-manifest and manifest participants of different caffeine—drinks (coffee or caffeinated drinks) which might have contributed to divergent effects in the investigated cohorts since no further information are provided in the dataset. However, and as a strength of this investigation, we used standardized data of large overall cohorts aiming to minimize such other potentially biasing factors. 

In summary, having analyzed the largest real-world cohort of pre- and motor-manifest HD mutation carriers with regard to caffeine-consumption, we found more psychiatric impairments in premanifest and manifest HD participants extensively consuming caffeine. Effects are potentially based on an exacerbation of psychiatric symptoms and neuronal-sympathetic stimulation or caused by changes in sleep-quality or sleep-time and fatigue. Data of motor-manifest HD patients suggest beneficial effects of caffeine consumption on disease manifestation and course of disease potentially due to regulation of pathophysiological functions via adenosine receptors or symptomatic effects such as alleviating some actions of neuroleptics. Further investigations, in particular, such as double-blinded interventional studies are necessary to validate findings and identify dosage or causal effects. Interventions should exclude potential other unknown biasing factors such as physical activity, pharmacological interventions, gender differences or chronic habitual influences. 

## Figures and Tables

**Table 1:** Baseline data of premanifest HD mutation carriers without caffeine consumption vs. participants drinking >3 cups of caffeine/day. As co-variables, we controlled for age CAG- repeat length and education.

	Premanifest HD No Caffeine Consumption (*n* = 841) M (SD)	Premanifest HD >3 Cups Caffeine/Day (*n* = 1901) M (SD)	*F*	*p*	*Part. Eta^2^*
Age (y)	37.1 (12.0)	41.9 (11.6)	99.35	<0.001	0.035
CAG high	42.7 (2.9)	42.2 (2.7)	19.54	<0.001	0.007
CAP-Score	316.6 (90.9)	340.9 (93.6)	39.88	<0.001	0.014
Calculated years to onset	18.3 (10.5)	16.2 (10.0)	29.97	<0.001	0.009
ISCED	3.9 (1.1) (*n* = 836)	3.8 (1.1) (*n* = 1894)	6.25	0.012	0.002
Sex (f/m) (%f/%m)	545/295 (64.9/35.1)	1012/888 (53.3/46.7)	32.04	<0.001	0.000
UHDRS TMS #	2.6 (4.3)	3.3 (4.4)	2.32	0.128	0.001
TFC +	12.7 (1.1) (*n* = 834)	12.7 (0.9) (*n* = 1890)	0.62	0.430	0.000
IS +	99.1 (4.1) (*n* = 836)	98.8 (3.9) (*n* = 1892)	0.00	0.956	0.000
SDMT +	50.1 (12.6) (*n* = 826)	47.9 (12.2) (*n* = 1884)	0.09	0.764	0.000
VFc +	21.2 (5.8) (*n* = 823)	20.9 (5.7) (*n* = 1882)	0.60	0.439	0.000
SCNT +	73.9 (15.0) (*n* = 825)	70.7 (14.8) (*n* = 1877)	8.22	<0.005	0.003
SWRT +	94.3 (18.9) (*n* = 825)	90.1 (18.5) (*n* = 1881)	12.44	<0.001	0.005
SIT +	43.9 (12.3) (*n* = 790)	41.7 (10.8) (*n* = 1780)	1.18	0.277	0.000
MMSE +	28.7 (1.8) (*n* = 622)	28.6 (1.7) (*n* = 1256)	0.07	0.786	0.000
PBA-Depression #	4.2 (5.9) (*n* = 833)	4.7 (6.2) (*n* = 1889)	4.23	<0.050	0.002
PBA-Irritability #	1.8 (3.6)	2.6 (3.8)	7.78	<0.050	0.003
PBA-Psychosis #	0.15 (1.1)	0.09 (0.8)	2.67	0.103	0.001
PBA-Apathy #	1.0 (2.5)	1.1 (2.4)	0.42	0.518	0.000
PBA-Executive function #	1.2 (3.1)	1.4 (3.3)	1.39	0.238	0.001
HADS-Anxiety #	5.4 (4.2) (*n* = 610)	5.8 (4.0) (*n* = 1241)	5.19	<0.050	0.003
HADS-Depression #	3.3 (3.4)	3.9 (3.7)	7.46	<0.050	0.004
SIS-Irritability #	4.5 (3.9)	5.6 (4.2)	21.46	<0.001	0.012
SIS-Outward irritability #	2.8 (2.4)	3.5 (2.5)	23.66	<0.001	0.013
SIS-Inward irritability #	1.7 (2.1)	2.1 (2.3)	9.67	<0.005	0.005

+, Higher scores = better performance; #, Higher scores = more impairment. Values highlighted (background colors): green = best performance; red = most impairment. Abbreviations: M, mean; SD, standard deviation; F, *F*-value; P, *p*-value; Part Eta^2^, partial Eta² effect size; y, years; CAG-high, Cytosine–Adenine–Guanine repeat length of the higher loaded allele; CAP-Score, CAG-Age Product- Index; ISCED, International Standard Classification of Education- educational level; UHDRS, Unified Huntington’s Disease Rating Scale including 3 subscales: TMS, Total motor score; TFC, Total functional capacity; IS, Independence scale; SDMT, Symbol digit modality test; VFc, Verbal fluency test (category); SCNT, Stroop color naming test; SWRT, Stroop word reading test; SIT, Stroop interference test; MMSE, Mini mental state examination; PBA, Problem Behaviours Assessment- Short Depression Scale with sub-scores; HADS, Hospital Anxiety and Depression Scale with sub-scores; SIS, Snaith Irritability Scale with sub-scores.

**Table 2:** Longitudinal analyses of motor, functional and cognitive parameters between groups. Data were analyzed using repeated measures analysis of variance between groups at baseline and three more follow up visits. Data depicted as mean performance levels (standard deviation) in groups and inter-subject effects. For co-variates, we used age, CAG and education.

	Premanifest HD No Caffeine Consumption (*n* = 197) M (SD)					Premanifest HD >3 Cups Caffeine/Day (*n* = 527) M (SD)					*F*	*p*	*Part. Eta^2^*
	BL	FU 1	FU 2	FU3	Δ *FU3-BL*	BL	FU1	FU2	FU3	Δ *FU3-BL*			
UHDRS- TMS #	2.8 (4.6) (*n* = 195)	3.5 (5.3)	4.1 (6.5)	4.7 (7.1)	1.9	3.3 (4.2) (*n* = 524)	4.5 (5.6)	5.3 (6.7)	6.1 (7.6)	2.8	0.99	0.321	0.001
TFC+	12.6 (1.2) (*n* = 197)	12.5 (1.2)	12.5 (1.3)	12.4 (1.4)	−0.2	12.7 (0.8) (*n* = 526)	12.6 (1.0)	12.3 (1.3)	12.3 (1.4)	−0.4	0.61	0.435	0.001
IS +	98.9 (4.4) (*n* = 197)	98.4 (4.7)	97.8 (5.7)	97.4 (6.4)	−1.5	99.0 (3.3) (*n* = 527)	98.1 (4.8)	97.1 (6.8)	96.4 (7.5)	−2.6	0.12	0.728	0.000
SDMT +	51.1(11.9) (*n* = 186)	51.7 (12.4)	52.3 (13.1)	52.1 (14.3)	1.0	48.9 (12.2) (*n* = 519)	49.1 (12.9)	49.1 (13.4)	48.5 (13.8)	−0.4	0.56	0.454	0.001
VFc +	21.3 (6.0) (*n* = 192)	21.9 (5.5)	22.3 (5.8)	21.5 (5.9)	0.2	21.3 (5.6) (*n* = 515)	21.4 (5.6)	21.3 (5.7)	21.5 (5.8)	0.2	0.06	0.805	0.000
SCNT +	72.6 (15.6) (*n* = 191)	73.3 (15.6)	74.2 (15.6)	73.1 (17.0)	0.5	72.1 (14.1) (*n* = 519)	71.7 (14.9)	71.0 (16.1)	70.9 (16.0)	−1.2	0.12	0.727	0.000
SWRT +	94.5 (19.5) (n = 191)	93.8 (19.5)	93.7 (20.2)	93.5 (20.9)	−1.0	90.6 (18.1) (n = 517)	89.9 (18.8)	88.3 (19.8)	88.0 (20.5)	−2.6	40.42	0.036	0.006
SIT +	43.8 (11.5) (*n* = 174)	44.5 (11.1)	44.1 (11.4)	44.4 (11.6)	0.6	42.8 (10.8) (*n* = 472)	43.4 (11.7)	43.6 (13.1)	42.8 (11.9)	0.0	0.61	0.433	0.001
MMSE +	28.8 (1.5) (*n* = 118)	29.0 (1.4)	29.0 (1.5)	28.9 (1.6)	0.1	28.6 (1.6) (*n* = 281)	28.5 (1.9)	28.7 (1.8)	28.7 (1.8)	0.1	0.86	0.353	0.002

+, higher scores = better performance; #, higher scores = more impairment; M, mean; SD, standard deviation; P, *p*-value; F, *F* value; Part Eta^2^, partial Eta² effect size; TMS, UHDRS total motor score; TFC, UHDRS total functional capacity; IS, UHDRS independence scale; SDMT: Symbol digit modality test; VFc, Verbal fluency test (Category); SCNT, Stoop color naming test; SWRT, Stroop word reading test; SIT, Stroop interference test; MMSE, Mini mental state examination.

**Table 3:** Manifest HD drinking >3 u caffeine/day vs. no caffeine consumption. Cross-sectional analysis of variance between groups with co-variables age, CAG-repeat length and education.

	Manifest HD No caffeine Consumption (*n* = 2243) M (SD)	Manifest HD >3 Cups Caffeine/Day (*n* = 4072) M (SD)	*F*	*P*	*Part. Eta^2^*
Age (y)	53.4 (13.8)	52.1 (12.1)	15.10	<0.001	0.002
CAG high	44.6 (4.6)	43.8 (3.6)	54.04	<0.001	0.009
CAP-Score	536.7 (108.0)	497.6 (93.8)	223.17	<0.001	0.034
ISCED	3.2 (1.4) (*n* = 2217)	3.4 (1.1) (*n* = 4064)	32.91	<0.001	0.005
Sex (f/m) (%f/m)	1202/1040 (53.6/46.4)	1955/2116 (48.0/52.0)	18.07	<0.001	0.000
Onset of symptoms					
Noticed by rater	45.4 (13.1) (*n* = 2145)	45.9 (11.7) (*n* = 3993)	72.35	<0.001	0.012
Noticed by subject	45.6 (13.3) (*n* = 2002)	45.9 (12.4) (*n* = 3800)	55.25	<0.001	0.009
Noticed by family	44.8 (13.3) (*n* = 2027)	45.2 (12.2) (*n* = 3664)	70.95	<0.001	0.012
HD-Diagnosis (y)	48.2 (13.6) (*n* = 2131)	48.6 (12.2) (*n* = 3945)	122.48	<0.001	0.020
UHDRS TMS #	45.1 (24.8) (*n* = 2168)	33.8 (18.8) (*n* = 4026)	205.18	<0.001	0.032
TFC+	6.9 (4.1) (*n* = 2185)	8.8 (3.3) (*n* = 4055)	227.22	<0.001	0.035
IS +	69.4 (23.6) (*n* = 2184)	80.4 (15.2) (*n* = 4049)	293.95	<0.001	0.045
SDMT +	19.5 (13.4) (*n* = 1848)	24.8 (12.1) (*n* = 3862)	111.92	<0.001	0.019
Verfct +	10.4 (5.9) (*n* = 2012)	12.9 (5.6) (*n* = 4000)	144.87	<0.001	0.024
SCNT +	26.7 (19.5) (*n* = 1971)	44.3 (16.5) (*n* = 3959)	139.47	<0.001	0.023
SWRT +	49.2 (26.3) (*n* = 1955)	58.3 (21.2) (*n* = 3951)	101.53	<0.001	0.017
SIT +	21.1 (12.3) (*n* = 1654)	24.5 (11.1) (*n* = 3489)	47.60	<0.001	0.009
MMSE +	23.5 (5.4) (*n* = 1418)	25.6 (3.8) (*n* = 2385)	118.32	<0.001	0.030
PBA-Depression #	5.1 (6.4) (*n* = 2097)	5.6 (6.7) (*n* = 4040)	1.63	0.202	0.000
PBA-Irritability #	3.4 (5.2)	3.7 (5.2)	1.87	0.172	0.000
PBA-Psychosis #	0.4 (1.9)	0.4 (1.8)	0.34	0.558	0.000
PBA-Apathy #	4.2 (5.0)	3.3 (4.2)	34.61	<0.001	0.006
PBA-Executive function #	4.0 (5.9)	3.4 (5.2)	11.29	<0.005	0.002
HADS-Anxiety #	5.8 (4.3) (*n* = 1168)	6.3 (4.3) (*n* = 2229)	4.18	<0.050	0.001
HADS-Depression #	6.6 (4.4)	6.1 (4.1)	10.79	<0.005	0.003
HADS-Irritability #	5.6 (4.7)	6.5 (4.7)	15.59	<0.001	0.005
SIS-Outward irritability #	3.4 (2.8)	3.9 (2.8)	19.81	<0.001	0.006
SIS-Inward irritability #	2.2 (2.5)	2.5 (2.5)	5.23	<0.050	0.002

+, higher scores = better performance; #, higher scores = more impairment. Values highlighted (background colors): green = best performance; red = most impairment. *Abbreviations:* M, mean; SD, standard deviation; P, *p*-value; F, *F* value; Part Eta^2^, Effect size; y, years; CAG, Cytosine–Adenine–Guanine repeat length; CAP-Score, CAG-Age Product- Index; ISCED, International Standard Classification of Education- educational level; HD, Huntington´s Disease; UHDRS, Unified Huntington’s Disease Rating Scale; TMS, UHDRS total motor score; TFC, UHDRS total functional capacity; IS, UHDRS independence scale; SDMT, Symbol digit modality test; Verfct, Verbal fluency test (category); MMSE: Mini mental state examination; SCNT, Stroop color naming test; SWRT, Stroop word reading test; SIT, Stroop interference test; PBA, Problem Behaviours Assessment- Short Depression Scale with sub-scores; HADS, Hospital Anxiety and Depression Scale with sub-scores; SIS, Snaith Irritability Scale with sub-scores.

**Table 4:** Longitudinal analyses of motor, functional and cognitive parameters between groups. Data were analyzed using repeated measures analysis of variance between groups at baseline and three more follow up visits. Data depicted as mean performance levels (standard deviation) in groups and intersubject effects. For co-variates, we used age, CAG and education.

	Manifest HD No Caffeine Consumption (*n* = 522) M (SD)					Manifest HD > 3/Cups Caffeine/Day (*n* = 1120) M (SD)					*F*	*p*	*Part. Eta^2^*
	BL	FU 1	FU 2	FU3	Δ *FU3-BL*	BL	FU1	FU2	FU3	Δ *FU3-BL*			
UHDRS TMS #	40.1 (22.8) (*n* = 515)	43.5 (23.2)	47.3 (23.7)	51.2 (23.9)	11.1	32.1 (18.1) (*n* = 1083)	35.3 (19.3)	38.6 (20.6)	42.0 (22.0)	9.9	29.29	<0.001	0.018
TFC +	7.8 (3.9)	7.1 (3.9)	6.5 (3.9)	5.8 (3.8)	−2.0	8.9 (3.2)	8.3 (3.3)	7.7 (3.5)	7.1 (3.6)	−1.8	22.32	<0.001	0.013
IS +	73.9 (20.5) (*n* = 520)	70.8 (20.8)	67.5 (21.5)	63.5 (22.5)	−10.4	80.9 (14.5) (*n* = 1117)	78.0 (15.5)	74.9 (16.8)	71.9 (18.5)	−10.0	37.49	<0.001	0.022
SDMT +	25.0 (12.0) (*n* = 341)	23.4 (12.3)	21.5 (12.8)	19.4 (12.7)	−5.6	28.3 (11.9) (*n* = 918)	27.1 (12.1)	25.6 (12.3)	23.4 (13.2)	−4.9	13.27	<0.001	0.010
VFc +	12.1 (5.3) (*n* = 426)	11.4 (5.2)	10.7 (5.3)	10.1 (5.4)	−2.0	13.9 (5.5) (*n* = 1027)	13.1 (5.5)	12.4 (5.6)	11.7 (5.7)	−2.2	15.72	<0.001	0.011
SCNT +	41.7 (16.1) (*n* = 407)	39.9 (16.3)	37.7 (16.3)	34.1 (17.2)	−7.6	47.3 (16.0) (*n* = 997)	44.9 (15.7)	42.6 (16.1)	40.0 (16.6)	−7.3	19.12	<0.001	0.013
SWRT +	57.8 (21.4) (*n* = 392)	53.2 (21.5)	50.3 (21.2)	45.1 (21.6)	−12.7	61.6 (19.8) (*n* = 987)	58.6 (20.6)	55.4 (21.0)	51.5 (21.3)	−10.1	8.15	<0.005	0.006
SIT +	25.8 (10.9) (*n* = 301)	23.7 (10.9)	22.4 (11.1)	20.5 (11.0)	−5.3	27.2 (11.0) (*n* = 780)	26.5 (10.6)	25.3 (10.9)	23.8 (11.3)	−3.4	7.79	<0.050	0.007
MMSE +	25.3 (3.5) (*n* = 253)	25.1 (3.9)	24.7 (4.3)	23.6 (5.0)	−1.7	26.4 (3.0) (*n* = 482)	26.3 (3.4)	25.9 (3.7)	25.4 (4.0)	−1.0	15.11	<0.001	0.020

+, higher scores = better performance; #, higher scores = more impairment. Values highlighted (background colors): green = best performance. Abbreviations: M, mean; SD, standard deviation; *P*, *p*-value; *F*, *F*-value; Part Eta^2^, partial eta² effect size; TMS, UHDRS total motor score; TFC, UHDRS total functional capacity; IS, UHDRS independence scale; SDMT, Symbol digit modality test; VFc, Verbal fluency test (category); SCNT, Stoop color naming test; SWRT, Stroop word reading test; SIT, Stroop interference test.

**Table 5:** Baseline data of family controls and genotype- negative HD without caffeine consumption vs. participants drinking >3 cups of caffeine/day. As co-variables we controlled for age.

	Family Control & Genotype Negative No Caffeine Consumption (*n* = 861) M (SD)	Family Control & Genotype Negative >3 Cups Caffeine/Day (*n* = 1528) M (SD)	*F*	*P*	*Part. Eta^2^*
Age (y)	45.3 (16.1)	48.3 (13.4)	23.946	<0.001	0.010
CAG high	19.9 (3.6)	20.5 (3.7)	10.076	<0.005	0.004
ISCED	3.7 (1.3) (*n* = 855)	3.8 (1.1) (*n* = 1527)	1.178	0.278	0.000
Sex (f/m) (%f/m)	553/309 (64.2/35.8)	817/713 (53.4/46.6)	26.286	<0.001	0.000
UHDRS TMS #	1.4 (3.3)	1.9 (3.2)	13.988	<0.050	0.003
TFC +	12.8 (1.1)	12.9 (0.5) (*n* = 1528)	17.352	<0.001	0.007
IS +	99.3 (3.8) (*n* = 862)	99.6 (2.3) (*n* = 1529)	8.805	<0.001	0.004
SDMT +	48.8 (13.2) (*n* = 852)	49.1 (11.6) (*n* = 1520)	7.740	<0.050	0.003
VFc +	21.4 (5.8) (*n* = 851)	21.8 (5.7) (*n* = 1523)	4.952	<0.050	0.002
SCNT +	73.1 (14.7) (*n* = 848)	73.8 (14.3) (*n* = 1514)	4.745	<0.050	0.002
SWRT +	94.1 (18.4) (*n* = 850)	94.3 (17.3) (*n* = 1517)	1.743	0.187	0.001
SIT +	41.9 (11.4) (*n* = 812)	41.7 (10.9) (*n* = 1419)	2.019	0.156	0.001
MMSE +	28.9 (1.5) (*n* = 667)	28.8 (1.6) (*n* = 1028)	.029	0.865	0.000
PBA-Depression #	3.3 (5.1) (*n* = 860)	3.6 (5.0) (*n* = 1527)	1.283	0.257	0.001
PBA-Irritability #	1.1 (2.6)	1.5 (2.9)	11.096	<0.005	0.005
PBA-Psychosis #	0.04 (0.4)	0.09 (0.8)	4.655	<0.050	0.002
PBA-Apathy #	0.4 (1.5)	0.5 (1.6)	1.761	0.185	0.001
PBA-Executive function #	0.7 (2.2)	0.7 (2.3)	.565	0.452	0.000
HADS-Anxiety #	5.0 (3.9) (*n* = 648)	5.5 (3.7) (*n* = 1158)	8.733	<0.005	0.005
HADS-Depression #	3.3 (3.4)	3.7 (3.3)	4.594	<0.050	0.003
HADS-Irritability #	4.0 (3.5)	4.6 (3.5)	15.726	<0.001	0.009
SIS-Outward irritability #	2.6 (2.2)	3.0 (2.2)	12.549	<0.001	0.007
SIS-Inward irritability #	1.4 (1.8)	1.7 (1.8)	10.286	<0.005	0.006

+: Higher scores = better performance; #: Higher scores = more impairment. Values highlighted (background colors): green= best performance; red = most impairment. Abbreviations: M, mean; SD, standard deviation; *P*, *p*-value; *F*, *F* value; Part Eta^2^: partial eta² effect size; y: years; CAG high, Cytosine–Adenine–Guanine repeat length of the higher loaded allele; ISCED, International Standard Classification of Education- educational level; UHDRS, Unified Huntington’s Disease Rating Scale; TMS, UHDRS total motor score; TFC, UHDRS total functional capacity; IS, UHDRS independence scale; SDMT, Symbol digit modality test; Verfct, Verbal fluency test (category); MMSE, Mini mental state examination; SCNT, Stroop color naming test; SWRT, Stroop word reading test; SIT, Stroop interference test; PBA, Problem Behaviours Assessment- Short Depression Scale with sub-scores; HADS, Hospital Anxiety and Depression Scale with sub-scores; SIS, Snaith Irritability Scale with sub-scores.

## Data Availability

The data that support the findings of this study are available from the corresponding author upon reasonable request.

## Supplementary Materials

The following supporting information can be downloaded at: https://www.mdpi.com/article/10.3390/biomedicines10061258/s1, Tables S1 and S2.
