# Supplementary material for: Divergent Effects of the Nonselective Adenosine Receptor Antagonist Caffeine in Pre-Manifest and Motor-Manifest Huntington’s Disease
Source: Biomedicines. 2022 May 27;10(6):1258. doi: 10.3390/biomedicines10061258 (PMC9219784; doi:10.3390/biomedicines10061258)
Supplement: Supplementary file 1 [file biomedicines-10-01258-s001.zip › biomedicines-1742028-supplementary.pdf]

Supplementary Table S1.

Years to calculated onset/ Variable	Premanifest HD no caffeine consumption (n = 841) M (SD)	Premanifest HD > 3 u caffeine/ day (n = 1901) M (SD)	*F*	*p*	*Part. Eta²*
**< 5 years**	**(*n* = 21)**	**(*n* = 67)**			
Years to onset	*4.5 (0.4)*	*4.4 (0.5)*	*1.538*	*.218*	*.018*
SDMT +	38.7 (10.2)	36.8 (11.2)	.474	.493	.006
VFc +	17.0 (6.3)	18.3 (5.6)	.740	.392	.009
SCNT +	59.0 (18.1)	58.7 (15.7)	.004	.949	.000
SWRT +	74.7 (20.5)	75.9 (16.5)	.068	.795	.001
SIT +	35.2 (13.5) (*n* = 17)	36.0 (11.7) (*n* = 63)	.056	.814	.001
MMSE +	28.1 (2.1) (*n* = 13)	27.0 (2.5) (*n* = 45)	2.476	.121	.042
**5- < 10 years**	**(*n* = 162)**	**(*n* = 531)**			
Years to onset	*7.8 (1.4)*	*7.7 (1.4)*	*.973*	*.324*	*.001*
SDMT +	42.7 (12.1)	42.4 (11.7)	.117	.732	.000
VFc +	18.9 (5.2)	19.6 (5.5)	1.581	.209	.002
SCNT +	67.5 (14.4)	65.5 (14.0)	2.398	.122	.004
SWRT +	87.5 (18.5)	84.5 (19.1)	3.196	.074	.005
SIT +	38.5 (12.4) (*n* = 149)	37.6 (10.4) (*n* = 494)	.831	.362	.001
MMSE +	28.3 (2.0) (*n* = 116)	28.2 (2.0) (*n* = 339)	.225	.635	.000
**10- < 15 years**	**(*n* = 205)**	**(*n* = 450)**			
Years to onset	*12.5 (1.4)*	*12.4 (1.5)*	*.136*	*.712*	*.000*
**SDMT +**	**49.2 (12.2)**	**47.0 (10.9)**	**4.949**	**<.050**	**.008**
VFc +	21.0 (6.0)	20.8 (5.6)	.272	.602	.000
SCNT +	72.8 (15.9)	71.3 (13.9)	1.423	.233	.002
**SWRT +**	**93.8 (19.5)**	**90.0 (16.6)**	**6.382**	**<050**	**.010**
**SIT +**	**44.1 (12.3) (*n* = 190)**	**41.8 (9.9) (*n* = 426)**	**5.874**	**<050**	**.009**
MMSE +	28.5 (2.1) (*n* = 147)	28.5 (1.8) (*n* = 304)	.008	.928	.000
**15- < 20 years**	**(*n* = 155)**	**(*n* = 321)**			
Years to onset	*17.5 (1.5)*	*17.1 (1.4)*	*6.836*	*.009*	*.014*
SDMT +	50.2 (10.5)	51.2 (10.5)	1.100	.295	.002
VFc +	22.0 (5.5)	21.7 (5.6)	.181	.671	.000

**SCNT +**	**76.5 (13.0)**	**72.4 (14.0)**	**9.618**	**<005**	**.020**
**SWRT +**	**98.1 (18.0)**	**93.0 (17.6)**	**8.720**	**<005**	**.018**
SIT +	44.6 (10.2) (*n* =148)	43.6 (10.5) (*n* = 297)	.860	.354	.002
MMSE +	28.7 (2.0) (*n* = 110)	28.8 (1.4) (*n* = 203)	.419	.518	.001
**> 20 years**	**(*n* = 298)**	**(*n* = 532)**			
Years to onset	*29.4 (9.3)*	*28.9 (9.0)*	*.593*	*.441*	*.001*
**SDMT +**	**55.5 (11.3)**	**53.4 (11.4)**	**6.386**	**<050**	**.008**
VFc +	22.5 (5.6)	22.5 (5.7)	.014	.905	.000
**SCNT +**	**77.8 (13.7)**	**75.8 (14.1)**	**3.927**	**<050**	**.005**
SWRT +	97.7 (17.3)	95.7 (17.5)	2.666	.103	.003
SIT +	46.9 (12.0) (*n* = 290)	45.3 (10.3) (*n* = 505)	3.703	.055	.005
MMSE +	29.1 (1.2) (*n* = 238)	29.0 (1.4) (*n* = 367)	.460	.498	.001

Supplementary Table S2.

	**Manifest HD**	**Manifest HD**	*F*	*p*	*Part. Eta²*
	**no caffeine consumption**	**> 3 u caffeine/ day**			
**Disease stage/ Variable**	**(*n* =2243) M (SD)**	**(*n* = 4072) M (SD)**			
**Stage 1 (TFC 13-11)**	**(*n* = 578)**	**(*n* = 1520)**			
TFC	*12.1 (0.8)*	*12.1 (0.8)*	*.828*	*.363*	*.000*
**SDMT +**	**30.0 (11.8)**	**31.8 (10.9)**	**10.250**	**<.005**	**.005**
**VFc +**	**14.9 (5.0)**	**15.8 (5.3)**	**9.736**	**<.005**	**.005**
SCNT +	51.9 (14.8)	53.2 (14.4)	3.247	.072	.002
SWRT +	70.7 (19.0)	70.5 (17.8)	.045	.833	.000
SIT +	28.9 (10.8) (*n* = 533)	29.9 (11.7) (*n* = 1389)	3.104	.078	.002
**MMSE +**	**26.9 (2.5) (*n* = 422)**	**27.2 (2.4) (*n* = 967)**	**4.601**	**<.050**	**.003**
**Stage 2 (TFC 10-7)**	**(*n* = 622)**	**(*n* = 1551)**			
TFC	*8.4 (1.1)*	*8.6 (1.1)*	*16.424*	*<.001*	*.008*
**SDMT +**	**21.4 (10.2)**	**23.5 (9.9)**	**17.793**	**<.001**	**.008**
**VFc +**	**11.7 (4.6)**	**12.6 (4.8)**	**16.171**	**<.001**	**.007**
**SCNT +**	**41.1 (14.8)**	**43.5 (14.1)**	**12.748**	**<.001**	**.006**
**SWRT +**	**54.9 (19.2)**	**56.7 (17.4)**	**4.529**	**<.001**	**.002**
SIT +	22.7 (10.5)	23.3 (9.7)	1.538	.215	.001

	(*n* = 533)	(*n* = 1372)			
**MMSE +**	**24.8 (3.4)**	**25.6 (3.0)**	**17.562**	**<.001**	**.012**
	**(*n* = 463)**	**(*n* = 933)**			
**Stage 3 (TFC 6-3)**	**(*n* = 560)**	**(*n* = 784)**			
TFC	*4.5 (1.1)*	*4.8 (1.1)*	*.29.796*	*<.001*	*.022*
**SDMT +**	**13.1 (9.0)**	**16.1 (9.0)**	**30.190**	**<.001**	**.025**
**VFc +**	**7.9 (4.2)**	**9.5 (4.5)**	**41.608**	**<.001**	**.031**
**SCNT +**	**29.4 (13.9)**	**33.4 (13.4)**	**27.101**	**<.001**	**.021**
**SWRT +**	**39.3 (18.9)**	**43.6 (17.2)**	**17.290**	**<.001**	**.014**
**SIT +**	**15.6 (9.4)**	**17.4 (9.2)**	**9.191**	**<.005**	**.009**
	**(*n* = 416)**	**(*n* = 614)**			
**MMSE +**	**21.7 (4.7)**	**23.2 (4.3)**	**22.23**	**<.001**	**.028**
	**(*n* = 361)**	**(*n* = 401)**			
**Stage 4 (TFC 2-1)**	**(*n* = 298)**	**(*n* = 176)**			
TFC	*1.6 (0.5)*	*1.6 (0.5)*	*.885*	*.347*	*.002*
**SDMT +**	**3.7 (5.5)**	**7.8 (7.2)**	**31.606**	**<.001**	**.097**
**VFc +**	**4.3 (3.1)**	**6.5 (3.7)**	**42.445**	**<.001**	**.097**
**SCNT +**	**15.1 (12.3)**	**22.8 (12.4)**	**35.021**	**<.001**	**.085**
**SWRT +**	**18.9 (16.0)**	**29.9 (17.7)**	**39.387**	**<.001**	**.096**
**SIT +**	**7.2 (7.1)**	**11.9 (7.8)**	**25.004**	**<.001**	**.089**
	**(*n* =156)**	**(*n* = 101)**			
**MMSE +**	**16.5 (6.0)**	**19.6 (5.6)**	**13.734**	**<.001**	**.058**
	**(*n* = 153)**	**(*n* = 72)**			
**Stage 5 (TFC 0)**	**(*n* = 174)**	**(*n* = 33)**			
TFC	*.00 (0.0)*	*.00 (0.0)*	.	.	.
SDMT +	.80 (2.9)	2.3 (5.6)	2.199	.141	.023
	(*n* = 83)	(*n* = 13)			
**VFc +**	**1.2 (2.6)**	**3.2 (2.3)**	**11.970**	**<.001**	**.093**
	**(*n* = 93)**	**(*n* = 26)**			
**SCNT +**	**3.9 (8.4)**	**13.3 (10.1)**	**23.288**	**<.001**	**.168**
	**(*n* = 92)**	**(*n* = 26)**			
**SWRT +**	**3.7 (9.0)**	**17.5 (15.6)**	**32.916**	**<.001**	**.223**
	**(*n* = 91)**	**(*n* = 26)**			
SIT +	3.2 (6.2)	5.8 (6.7)	1.677	.201	.032
	(*n* = 39)	(*n* = 14)			
MMSE +	11.9 (7.0)	12.3 (7.5)	.031	.862	.001
	(*n* = 34)	(*n* = 12)			
